# PROMOTING KNOWLEDGE OF COGNITIVE DYSFUNCTION IN MINORITY COMMUNITY-DWELLING OLDER ADULTS

**DOI:** 10.1093/geroni/igz038.3010

**Published:** 2019-11-08

**Authors:** Koshy Alexander, Ruth Manna, Natalie Gangai, Rosario Costas Muniz, Beatriz Korc-Grodzicki

**Affiliations:** Memorial Sloan Kettering Cancer Center, New York, United States

## Abstract

Minorities and older adults represent an intersection of populations that are particularly vulnerable to suboptimal care. The US population is aging and growing more diverse. Dementia afflicts minority populations more than Caucasians. Many people in the community with mild cognitive impairment (MCI) and Alzheimer’s disease do not recognize cognitive, functional or behavioral impairment as abnormal. Several barriers to treatment exist within both the older and minority populations. The Health Resources and Services Administration (HRSA), is the primary Federal agency for improving health and achieving health equity. Queens County in New York City is a multicultural, ethnically diverse urban area with 48% of its population being foreign born. Funded by a grant from HRSA, in collaboration with the South Asian Council for Social Services (SACSS), the Geriatric Resource Interprofessional Program (GRIP) at Memorial Sloan Kettering Cancer Center (MSKCC) developed and conducted educational lectures on “Memory Loss & Dementia” at 7 different community centers in Queens. Between November 2015 and January 2019 eight sessions were conducted. A total of 182 people attended. Their mean age was 68, 59% were women. 71% were born in South Asia. Consecutive interpretation of the lecture was performed; the written materials were translated beforehand. Pre and post-test questionnaires were administered to measure understanding of the topic, and qualitative comments collected. The developmental process, results of the study and challenges we faced in developing and applying the educational initiative will be presented.

